# Cudratricusxanthone O Inhibits H_2_O_2_-Induced Cell Damage by Activating Nrf2/HO-1 Pathway in Human Chondrocytes

**DOI:** 10.3390/antiox9090788

**Published:** 2020-08-25

**Authors:** Eun-Nam Kim, Hyun-Su Lee, Gil-Saeng Jeong

**Affiliations:** College of Pharmacy, Keimyung University, 1095 Dalgubeol-daero, Daegu 42601, Korea; enkimpharm@gmail.com (E.-N.K.); hyunsu.lee@kmu.ac.kr (H.-S.L.)

**Keywords:** cudratrixanthone O, reactive oxygen species, nuclear transcription factor erythroid-2-like factor 2, hemeoxygenase-1, apoptosis

## Abstract

Osteoarthritis (OA) is a common joint degenerative disease induced by oxidative stress in chondrocytes. Although induced-heme oxygenase-1 (HO-1) has been found to protect cells against oxygen radical damage, little information is available regarding the use of bioactive compounds from natural sources for regulating the HO-1 pathway to treat OA. In this study, we explored the inhibitory effects of cudratricusxanthone O (CTO) isolated from the *Maclura tricuspidata* Bureau (*Moraceae*) on H_2_O_2_-induced damage of SW1353 chondrocytes via regulation of the HO-1 pathway. CTO promoted HO-1 expression by enhancing the translocation of nuclear factor erythroid 2-related factor 2 (Nrf2) into the nucleus without inducing toxicity. Pretreatment with CTO-regulated reactive oxygen species (ROS) production by inducing expression of antioxidant enzymes in H_2_O_2_-treated cells and maintained the functions of H_2_O_2_-damaged chondrocytes. Furthermore, CTO prevented H_2_O_2_-induced apoptosis by regulating the expression of anti-apoptotic proteins. Treatment with the HO-1 inhibitor tin-protoporphyrin IX revealed that these protective effects were exerted due to an increase in HO-1 expression induced by CTO. In conclusion, CTO protects chondrocytes from H_2_O_2_-induced damages—including ROS accumulation, dysfunction, and apoptosis through activation of the Nrf2/HO-1 signaling pathway in chondrocytes and, therefore, is a potential therapeutic agent for OA treatment.

## 1. Introduction

Osteoarthritis (OA) is a chronic joint degenerative disease that affects normal movements due to loss of articular cartilage, particularly in older adults [1,2]. OA is accompanied by decomposition and destruction of the mesochondrium and cartilage, as well as synovial inflammation, but the main pathological reasons include oxidative stress, aging, and expression of inflammation-related genes [3]. A previous study showed that the degradation of the extracellular matrix (ECM) by the inflammatory response is essential for OA progression, and the damaged joint tissues produce severe cytokines and ECM degradation by-products [4]. Chondrocytes play an important role in maintaining the function of the joints and can generate tissue ECM—including collagen II and proteoglycan—to maintain tissue homeostasis and joint movement [5]. Reactive oxygen species (ROS)—such as hydrogen peroxide (H_2_O_2_)—are crucial modulators of the redox-sensitive cell signaling pathway, and involved in biological processes—such as host defense, oxygen sensing, proliferation, and apoptosis. However, from a pathological point of view, the overproduction of ROS is associated with inflammation, atherosclerosis, diabetes, high blood pressure, tumor formation, and OA [6]. Therefore, suppressing cell damage to chondrocytes by regulating ROS production in osteoarthritis is an important treatment strategy.

To eliminate cell damage caused by excessive ROS production, most cells, including chondrocytes, have endogenous defense strategies that protect cells from oxidative stress through the nuclear factor erythroid-2-related factor 2 (Nrf2) pathway [7,8]. The defense system activated by Nrf2 leads to induction of superoxide dismutase (SOD), glutathione peroxidase (GPx), glutathione (GSH), and heme oxygenase-1 (HO-1) [9]. Heme oxygenases (HOs) are a group of enzymes that catalyze heme breakdown, and four metabolites have been identified: iron, carbon monoxide (CO), and biliverdin. Three types of HO have been discovered, including HO-1, HO-2, and HO-3. HO-1 plays a key role in the defense mechanism against oxidative damage [10,11,12]. Although activation of Nrf2 and HO-1 as endogenous defense mechanisms in chondrocytes is important, little is known about whether bioactive small molecules isolated from natural products promote the Nrf2/HO-1 pathway to defend cells from oxidative damage.

*Maclura tricuspidata* Bureau (*Moraceae*) is a deciduous broad-leaved tree that is common in China, Korea, and Japan. It has been used in Korean traditional medicine to treat inflammation, gastritis, cancer, and liver damage [13,14,15]. The major components of *M. tricuspidata* are xanthones, flavonoids, isoflavonoids, and benzylated flavonoids. Among them, prenylated xanthones exhibit antioxidative, anti-inflammatory, antiatherosclerotic, and neuroprotective activities [16,17,18]. Moreover, prenylated xanthones have shown anti-inflammatory effects in RAW264.7 cells stimulated with lipopolysaccharides (LPS) by inhibiting the expression of pro-inflammatory mediators through HO-1 expression [19]. However, despite these various biological activities, prenylated xanthones have not been studied for OA. Previous studies have shown that hypoxia or inflammatory induction of SW1353 chondrocytes stimulated by IL-1β, monosodium iodoacetate (MIA), and H_2_O_2_ is known as a representative in vitro model for OA studies [20,21,22,23]. In this study, we investigated the role of prenylated xanthones, cudratricusxanthone O (CTO), isolated from *M. tricuspidata*, on the suppression of H_2_O_2_-induced cell damage by promoting the Nrf2/HO-1 pathway in SW1353 cells.

## 2. Materials and Methods

### 2.1. Chemicals and Reagents

Dulbecco’s Modified Eagle Medium (DMEM), fetal bovine serum (FBS), penicillin, and streptomycin were purchased from Welgene Inc. (Korea). 3-(4,5-Dimethylthiazol-2-yl)-2,5-diphenyltetrazoliumbromide (MTT) was purchased from Amresco Inc. (Solon, OH, USA). The primary antibodies of Nrf2, HO-1, β-actin, Bcl-2, superoxide dismutase (SOD) and catalase (CAT) were purchased from Santa Cruz Biotechnology Inc. (Santa Cruz, CA, USA), Bax and Caspase-3 were purchased from Cell Signaling Technology (Danvers, MA, USA). Hydrogen peroxide solution (H_2_O_2_), Protoporphyrin IX (SnPP), Cobalt protoporphyrin (CoPP) and DCF-DA (2′, 7′-dichloroflourescin diacetate) were bought from Sigma Aldrich (St. Louis, MO, USA). RIPA buffer and ECL Western blotting detection reagents were purchased from Fisher Scientific Inc. (Waltham, MA, USA).

### 2.2. Plant Materials and Isolation of Compounds

A voucher specimen (accession number KMU 002019-0116) was deposited at the College of Pharmacy, Keimyung University, Daegu, Korea. Cudratrixanthone O (CTO) was isolated from the bark extract of *M. tricuspidata*, and the structure of CTO was identified using nuclear magnetic resonance (NMR) and electrospray ionization mass spectrometry (ESIMS) compared with previously reported literature [24].

### 2.3. Cell Culture

The human chondrosarcoma cell line SW1353 was purchased from the American Type Culture Collection (ATCC, Manassas, VA, USA) and cultured in Dulbecco’s modified Eagle medium (DMEM) (Welgene, Gyeongsangbuk-do, Korea) containing 10% (v/v) fetal bovine serum, 10 μg/mL streptomycin, and 100 U/mL penicillin (Gibco BRL, Grand Island, NY, USA) in incubated on at 37 °C in 5% CO_2_.

### 2.4. Cell Viability and Coefficient Assays

SW1353 cells (5 × 10^3^ cells/well) were seeded in 96-well plates for 24 h, and cultured with or without CTO (1, 2, 5 μM) for 24 h. Then, 50 μL of MTT (5 mg/mL in PBS, Sigma-Aldrich) was treated to each well for 4 h. Four hours later, supernatant was aspirated, and 150 μL of DMSO was added to each well. The absorbance values were measured at 540 nm on a microplate reader (TECAN, Austria), and for confluency assays cells were seeded in 24-well plates for 24 h, after then, with CTO (1, 2, 5 μM), and the cells counted with Incucyte^®^ Live-Cell analysis systems (Göttingen, Germany).

### 2.5. Western Blot Analysis

Western blot analysis was performed to examine the expression levels of indicated proteins in SW1353 chondrocytes. Cells were lysed in RIPA buffer containing protease inhibitors and centrifuged at 14,000 rpm for 30 min and quantitate by Bradford assay using a Bio-Rad Bradford assay reagent (Hercules, CA, USA). Then, proteins were separated using 8–12% SDS/polyacrylamide gel electrophoresis and transferred on to PVDF membranes, After blocking with TBS-T buffer containing skim milk (5%), The membranes were incubated with the primary antibodies overnight at 4 °C, after with a secondary antibody. PVDF membrane was detected with Healthcare Life Science ECL-plus (Tokyo, Japan), the images were taken by ImageQuant LAS 4000 (GE Healthcare Life Science, Tokyo, Japan). The expressional value of cytosolic proteins was normalized to the intensity level of β-actin and proteins compared with the untreated cells (control) using image J software.

### 2.6. Cytosolic and Nuclear Protein Extraction

SW1353 cells were seeded at 5 × 10^5^ cells/mL in a 6-well plate. The harvested cells were then lysed on ice for 20 min with radioimmunoprecipitation assay (RIPA) buffer (Thermo Fisher Scientific, Waltham, MA, USA) and the isolated cytoplasm and nuclei were removed using the NE-PER nuclear and cytoplasmic extraction reagent kit (Pierce Biotechnology, Rockford, IL, USA) according to the manufacturer’s instructions.

### 2.7. Measurement of ROS Generation

The production of intracellular ROS was assessed using a cell-permeable fluorogenic probe, 2′, 7′-dichlorodihydrofluorescein diacetate (DCF-DA). SW-1353 cells were seeded in 6-well plate at 1 × 10^5^ cells/well for 24 h, and after pretreated with different concentrations of CTO for 6 h and then cultured for 2 h in the presence or absence of 0.5 mM H_2_O_2_. Then, the cells were washed twice with PBS to and DCF-DA was incubated in a dark place at 37 °C for 20 min. After 20 min, cells were washed with PBS and fixed with 4% paraformaldehyde (pH 7.4) for 20 min. After observation, the ROS was detected by a fluorescence Olympus IX microscope 71-F3 2PH (Tokyo, Japan).

### 2.8. RT-qPCR Analysis

After treatment, total RNA was extracted from the SW-1353 cells using TRIzol/chloroform reagent (Bioneer, Korea) according to the manufacturer’s instructions. Total RNA was transcribed into cDNA by PrimeScript-RT reagent kit, then the cDNA was amplificated by the SYBR Premix Ex Taq (Sangon). The cycling conditions were 40 cycles at 50 °C for 2 min, 95 °C initial denaturation for 10 min, 95 °C denaturation for 15 s, and 60 °C annealing for 30 s. The mRNA encoding each target was measured using real-time PCR and GAPDH was used as the housekeeping gene. The cycle threshold (Ct) value of the target gene was normalized to GAPDH. The primers and amplification products of each gene used in this study are shown in Table 1.

### 2.9. Annexin V-FITC/PI Apoptosis Assay

SW-1353 cells were seeded in six-well plates for 24 h, then pretreated with CTO (0, 1, 2, 5 μM) for 6 h. The positive control group and the CTO-treated groups were then exposed to H_2_O_2_ to a final concentration of 0.5 mM for 2 h. Cells were collected by centrifugation and washed twice with PBS, and apoptotic incidence was analyzed by the Annexin V-FITC/PropidiumIodide (PI) detection kit (BD Biosciences, San Diego, CA, USA) according to the manufacturer’s instructions. The rate of apoptosis was analyzed using a Incucyte^®^ Live-Cell analysis systems.

### 2.10. Statistical Analysis

Each experiment was performed in triplicate and expressed as mean value and standard deviation. Statistical analysis was conducted using SPSS Statistics 19.0 software. Differences among groups were analyzed by one-way analysis of variance (ANOVA) followed by Tukey’s test or Student’s *t*-test. *p* < 0.05 were considered to indicate statistical significance.

## 3. Results

### 3.1. CTO Is Not Cytotoxic to SW1353 Chondrocytes

**Figure 1:**
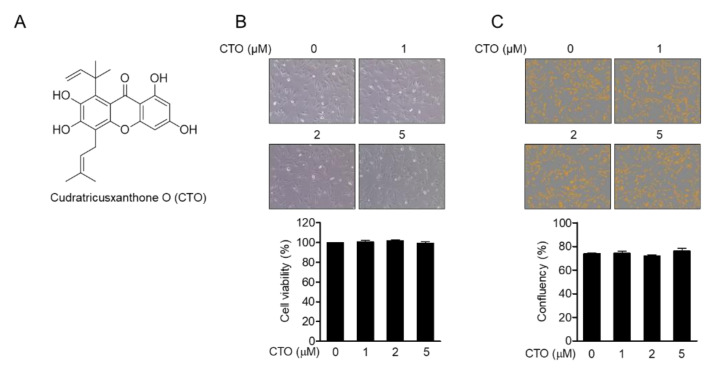
Cudratricusxanthone O (CTO) is not cytotoxic to SW1353 chondrocytes. (**A**) The chemical structure of CTO. (**B**) SW1353 chondrocytes were seeded at a density of 1 × 10^4^ cells/well and treated with CTO at the indicated concentrations (0–5 μM) for 24 h, then after CTO cytotoxicity was evaluated by 3-(4,5-Dimethylthiazol-2-yl)-2,5-diphenyltetrazoliumbromide (MTT) assay. (**C**) The confluency of cells was determined using IncuCyte imaging system. The Student’s *t*-test was used for statistical analysis.

To investigate whether CTO (Figure 1A) showed cytotoxicity in chondrocytes, SW1353 chondrocytes were treated with 0, 1, 2, and 5 μM of CTO for 24 h, and cell viability was examined by MTT analysis. No cytotoxicity was observed in the CTO-treated cells, and no morphological change in CTO-treated SW1353 cells (Figure 1B). Moreover, treatment with CTO (0–5 μM) did not affect cellular confluency in SW1353 cells (Figure 1C). CTO showed no statistically significant changes in cytotoxicity or cellular confluency.

### 3.2. CTO Induces HO-1 Expression by Promotion of Nrf2 Translocation

**Figure 2:**
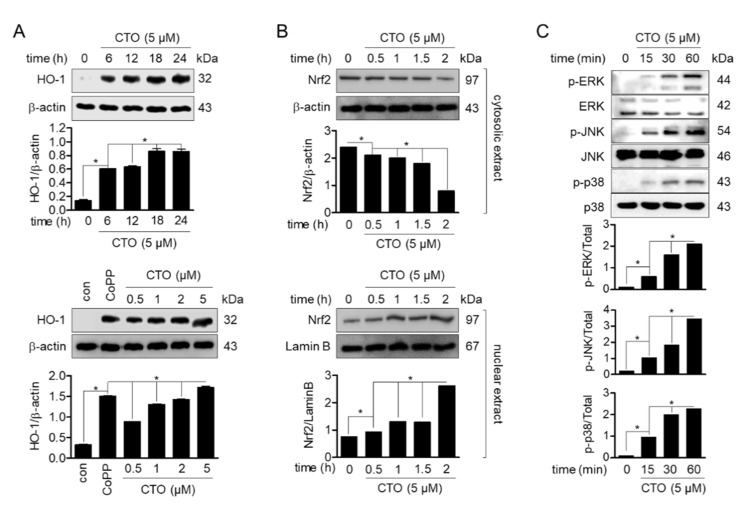
CTO induces heme oxygenase-1 (HO-1) expression by the promotion of Nrf2 translocation. (**A**) The cells (5 × 10^5^ cells/mL) were treated with 5 μM for the indicated time (0, 6, 12, 18, 24 h) or with the indicated concentrations of CTO (0.5, 1, 2, and 5 μM) and cobalt protoporphyrin (CoPP) (20 μM) for 18 h. The induced HO-1 expression was detected by Western blot analysis. (**B**) The translocation of Nrf2 was analyzed by Western blot analysis from cells treated with 5 μM of CTO for the indicated time. (**C**) The cells were treated with 5 μM CTO for the indicated times (0–60 min) and phosphorylation of ERK1/2, JNK and p38 were determined by Western blot analysis. ^*^ *p* < 0.05 was considered significant differences between groups are indicated.

Nrf2 and HO-1, the major downstream antioxidant signaling pathway, play a major role in regulating oxidative stress. Therefore, the protein expression of HO-1 was measured by Western blot to confirm that CTO activates the Nrf2/HO-1 signaling pathway. After treatment with 5 μM of CTO in SW1353 cells, the expression of HO-1 was induced in a time- and dose-dependent manner. HO-1 was expressed in cells treated with CTO for 6 h and showed the highest expression at 18 h (Figure 2A, top). Concentration-dependent experiments showed that treatment with CTO promoted HO-1 expression in a dose-dependent manner (Figure 2A, bottom). Western blot was used to verify whether Nrf2 translocation into the nucleus was involved in HO-1 expression by CTO. Dose-dependent treatment with 5 μM CTO reduced Nrf2 in the cytosol but enhanced its expression in the nucleus (Figure 2B). HO-1 expression is induced by the activation of MAPK, of which ERK1/2, JNK, and p38 act as upstream regulators of the Nrf2 cascade. We investigated whether CTO promotes Nrf2 translocation and HO-1 induction through activation of the MAPK pathway. Western blotting was performed on SW1353 cells treated with CTO 5 μM in a time-dependent manner for 15, 30, and 60 min. Gradually enhanced phosphorylation of MAPKs was observed after CTO treatment (Figure 2C). These results suggested that CTO was involved in Nrf2 translocation and HO-1 expression by activation of MAPKs in SW1353 cells.

### 3.3. CTO Suppresses ROS Production by Inducing SOD and CAT in SW1353 Cells

**Figure 3:**
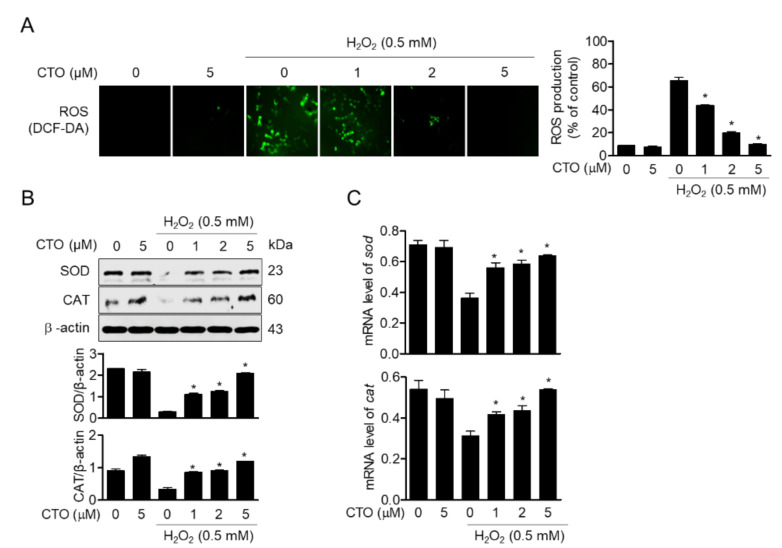
CTO suppresses ROS production by inducing superoxide dismutase (SOD) and catalase (CAT) in SW1353 cells. (**A**) The cells were pre-treated with the indicated concentrations of CTO for 6 h, and then stimulated with or without 0.5 mM H_2_O_2_ for 2 h. The cells were incubated at 37 °C in the dark for 20 min with culture medium containing 2 μM 2′, 7′-dichloroflourescin diacetate (DCF-DA) to monitor reactive oxygen species (ROS) production. The degree of ROS production was measured by fluorescence microscope. (**B**) The expression of SOD and CAT proteins were measured by Western blot analysis from cells pre-treated with CTO and stimulated by 0.5 mM H_2_O_2_. (**C**) The mRNA level of these genes was measured by real-time PCR. ^*^ *p* < 0.05 was considered significant compared to only H_2_O_2_ treat group.

It has been reported that treatment with H_2_O_2_ of chondrocytes produces ROS, which leads to cell apoptosis. To evaluate the effect of CTO on the production of ROS from cells treated with H_2_O_2_ in chondrocytes, the production of ROS and the expression of antioxidant enzymes such as SOD and CAT were evaluated in H_2_O_2_-stimulated SW1353 cells. In DCF-DA fluorescence staining, H_2_O_2_-stimulated cells showed an increase in ROS, but pretreatment with dose-dependent CTO reduced ROS production (Figure 3A). Expression levels of antioxidant proteins SOD and CAT were inhibited by H_2_O_2_ treatment but significantly protected by CTO treatment (Figure 3B). Furthermore, the effect of CTO on mRNA levels of SOD and CAT was evaluated by real-time PCR. We confirmed that expression levels of both antioxidant protein and mRNA were protected (Figure 3C). These results suggest that CTO regulates ROS production by preventing H_2_O_2_-induced apoptosis and retaining the expression of antioxidant proteins in chondrocytes.

### 3.4. CTO Up-Regulates Chondrocytes-Specific Genes but Inhibits the Expression of MMPs in H_2_O_2_ Treated SW1353 Cells

**Figure 4:**
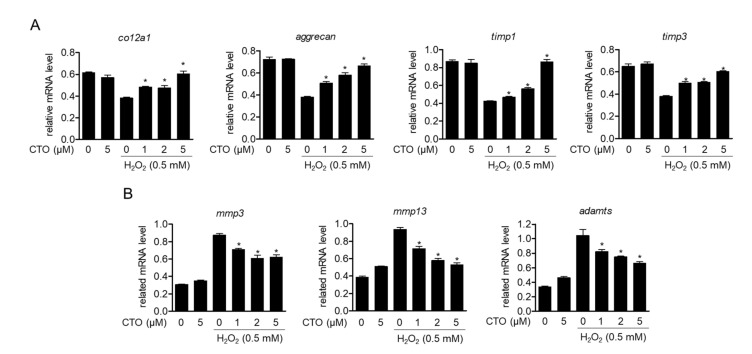
CTO up-regulates chondrocytes-specific genes but inhibits the expression of *mmps* in H_2_O_2_ treated SW1353 cells. (**A**,**B**) SW1353 cells were pre-treated with the indicated concentration of CTO (0, 1, 2, and 5 μM) for 6 h, and then incubated with 0.5 mM H_2_O_2_ for 2 h. The mRNA levels of cartilage-specific core genes and *timps* (**A**) and *mmps* genes (**B**) were determined by real-time PCR analysis. The results were normalized to *gapdh* expression. ^*^ *p* < 0.05 was considered significant compared only to H_2_O_2_ treat group.

Accumulating evidence suggests that H_2_O_2_-induced ROS production degrades the function of chondrocytes. Cartilage-specific genes such as *col2a1* and *aggrecan* play an essential role in the creation and maintenance of cartilage tissue, and proteolytic enzymes such as MMPs induce cartilage tissue loss. Therefore, to explore whether CTO pretreatment prevents functional loss of chondrocytes by H_2_O_2_, the mRNA levels of essential genes for cartilage tissue formation (*col2a1, aggrecan*), *timps, mmps*, and *adamts* were measured using real-time PCR in H_2_O_2_-treated SW1353 chondrocytes. We found that the mRNA levels of *col2a1, aggrecan*, *timp1*, and *timp3* were suppressed by H_2_O_2_, but protected in a concentration-dependent manner by CTO (Figure 4A). Moreover, we confirmed that the mRNA levels of *mmps* and *adamts* (a group of secreted proteinases and proteolytic enzymes), which suppressed the functions of chondrocytes, were increased by H_2_O_2_ treatment but significantly decreased by CTO treatment (Figure 4B). These results suggest that CTO protects the function of chondrocytes by upregulating essential genes for cartilage tissue formation as well as downregulating *mmps* and *adamts* upon H_2_O_2_ treatment.

### 3.5. CTO Inhibits Apoptotic Pathway Induced by Treatment with H_2_O_2_ in SW1353 Cells

**Figure 5:**
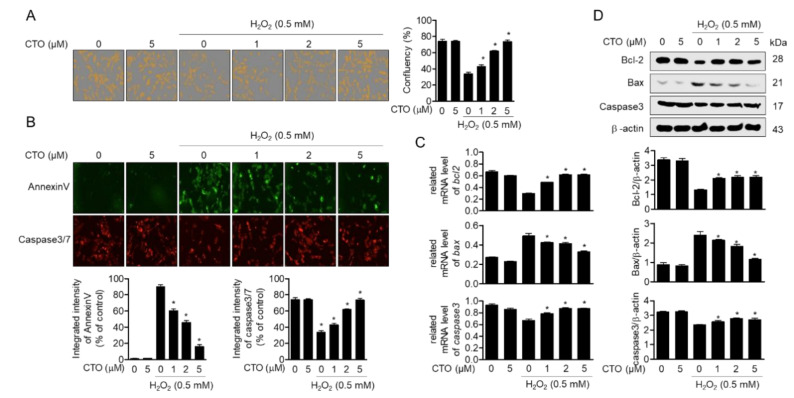
CTO inhibits the apoptotic pathway induced by treatment with H_2_O_2_ in SW1353 cells. (**A**, **B**) The cells were pre-treated with or without the indicated concentration of CTO (0, 1, 2, and 5 μM) for 6 h and then incubated with 0.5 mM H_2_O_2_ for 2 h. The confluency of cells was measured by IncuCyte imaging system (**A**), and apoptotic cells were evaluated by staining with Annexin V and caspase-3/7 (**B**). (**C**, **D**) The mRNA levels of *bcl-2*, *bax* and *caspase3* were measured by real-time PCR (**C**) and protein expressions were confirmed by Western blot analysis (**D**). The results were normalized to *gapdh* (**C**) or β–actin (**D**) expression. ^*^ *p* < 0.05 was considered significant compared to only H_2_O_2_ treat group.

To evaluate whether pretreatment with CTO of chondrocytes prevents H_2_O_2_-induced apoptosis, we measured the confluency of H_2_O_2_-stimulated cells. Cellular confluency revealed that pretreatment with CTO protected cells from the cytotoxicity of H_2_O_2_ in chondrocytes in a concentration-dependent manner (Figure 5A). To confirm whether pretreatment with CTO prevents cells to undergo apoptosis, Annexin V and caspase 3/7 from SW1353 cells pretreated with CTO and stimulated with H_2_O_2_ were detected by IncuCyte imaging system. In the CTO-pretreated cells, the previously increased intensity of Annexin V by H_2_O_2_ was then significantly reduced, and the previously decreased intensity of caspase 3/7 by H_2_O_2_ was then enhanced. (Figure 5B). To examine whether CTO pretreatment affected the mRNA levels of anti-apoptotic genes such as *bcl2* and *caspases3* or pro-apoptotic genes including *bax* in H_2_O_2_-treated conditions, we performed real-time PCR for SW1353 cells incubated with the indicated condition. The expression of *bcl2* and *caspases3* was reduced by H_2_O_2_ treatment, but retained by CTO pretreatment. On the other hand, the enhanced expression of the pro-apoptotic gene *bax* by H_2_O_2_ treatment decreased in a concentration-dependent manner by CTO pretreatment (Figure 5C). Protein levels of these genes were confirmed through Western blotting (Figure 5D). These results suggest that CTO pretreatment effectively prevents chondrocytes from H_2_O_2_-induced apoptosis by regulating the expression of apoptosis-related genes.

### 3.6. Upregulated HO-1 by Pre-Treatment with CTO Protects SW1353 Cells from ROS Production Induced by H_2_O_2_

**Figure 6:**
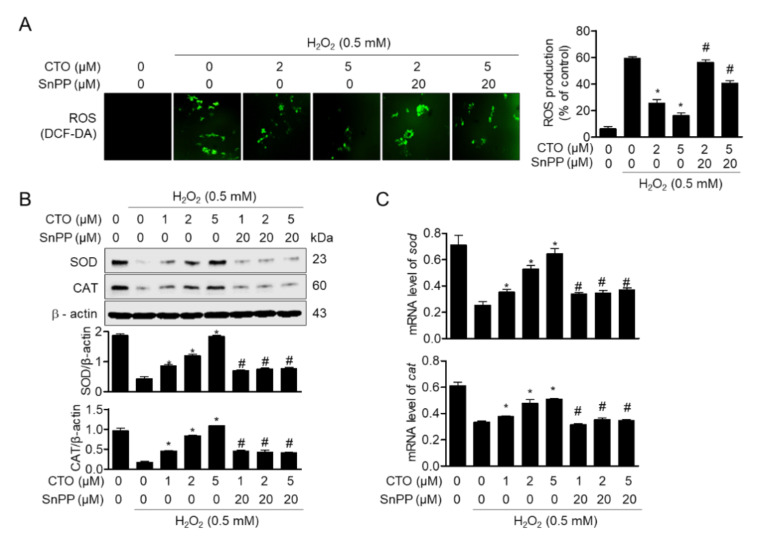
Upregulated HO-1 by pre-treatment with CTO protects SW1353 cells from ROS production induced by H_2_O_2_. (**A**) SW1353 cells were seeded at a density of 5 × 10^4^ cell/well and pre-treated with or without 20 μM Protoporphyrin IX (SnPP) for 1 h. Then cells were treated with the indicated concentrations of CTO (0, 1, 2, and 5 μM) for 6h, and incubated with 0.5 mM of H_2_O_2_ for 2h. The cells were incubated with culture medium containing 20 μM DCF-DA to monitor ROS production at 37 °C in the dark for 20 min. The images were obtained by fluorescent microscope system. (**B**, **C**) Protein expressions and mRNA levels of *sod* and *cat* were detected by Western blot analysis (**B**) and real-time PCR (C) from cells cultured with the indicated conditions. The results were normalized with β–actin (**B**) or *gapdh* (**C**) expression. ^*^ *p* < 0.05 vs. only H_2_O_2_ treat group; ^#^ *p* < 0.05 vs. only CTO treated group.

In Section 3.2, we showed that CTO effectively induces HO-1 expression by promoting Nrf2 translocation in SW1353 cells. We next investigated the effect of CTO on ROS production and antioxidant enzymes induced by H_2_O_2_ treatment in suppressing the expression of HO-1 by treatment with tin protoporphyrin IX (SnPP), an inhibitor of heme oxygenase enzyme. CTO treatment suppressed ROS generated by H_2_O_2_ stimulation in a concentration-dependent manner; however, the inhibitory effect of CTO on ROS production was reversed in the presence of SnPP (Figure 6A). Moreover, the recovery of SOD and CAT expression by pretreatment with CTO in H_2_O_2_-treated cells was inhibited in the SnPP-treated group (Figure 6B) and mRNA (Figure 6C). These results suggested that HO-1 induced by CTO pretreatment in H_2_O_2_-stimulated SW1353 cells is involved in the regulation of ROS production and recovery of antioxidant enzymes.

### 3.7. Induced HO-1 by CTO Regulates Chondrocytes-Specific Genes and Expression of mmps in H_2_O_2_ Treated SW1353 Cell

**Figure 7:**
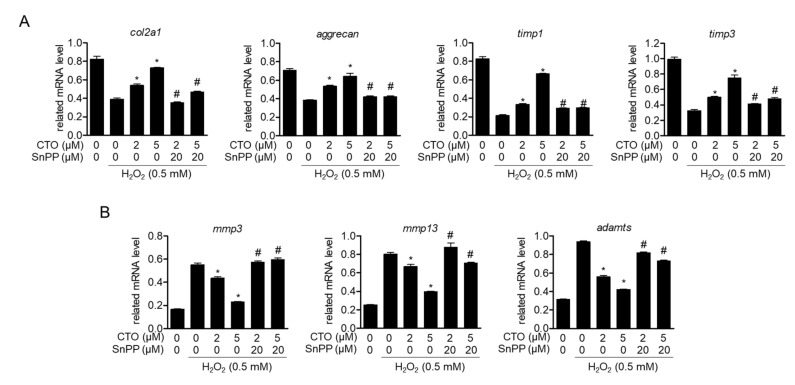
Induced HO-1 by CTO regulates chondrocytes-specific genes and expression of MMPs in H_2_O_2_ treated SW1353 cells. (**A**, **B**) SW1353 cells were pre-treated with or without 20 μM SnPP for 1h. Then cells were treated with the indicated concentration of CTO (2 or 5 μM) for 6 h, and incubated with 0.5 mM H_2_O_2_ for 2 h. The mRNA levels of cartilage-specific core genes and *timps* (**A**) and *mmps* genes (**B**) were determined by real-time PCR analysis. The results were normalized to *gapdh* expression. ^*^ *p* < 0.05 vs. only H_2_O_2_ treat group; ^#^ *p* < 0.05 vs. only CTO treated group.

We evaluated the effects of CTO on the specific genes and its function regulation of chondrocytes. Therefore, we additionally evaluated whether HO-1 expression by pretreatment with CTO protects chondrocyte-specific genes and functions in SW1353 cells in H_2_O_2_-treated conditions. CTO dose-dependently recovered the mRNA levels of *col2a1, aggrecan, timp1* and *timp3*; however, SnPP-treated cells did not show recovery of *col2a1, aggrecan, timp1* and *timp3* in H_2_O_2_-treated conditions (Figure 7A). We subsequently evaluated the effect of HO-1 expression by CTO treatment in H_2_O_2_-stimulated SW1353 cells on the mRNA levels of *mmps* and *adamts*, components of the extracellular matrix. We confirmed that the enhanced mRNA levels of *mmp3*, *mmp13*, and *adamts* by H_2_O_2_ were significantly blocked by CTO treatment, but recovered by treatment with SnPP (Figure 7B). These results suggest that the CTO-induced HO-1 protects SW1353 cells against dysfunction caused by H_2_O_2_ treatment, such as restoring cartilage-specific core proteins and inhibiting proteolytic enzymes.

### 3.8. Enhanced HO-1 by CTO Shows Protective Effect from Apoptosis Induced by H_2_O_2_ in SW1353 Cells

**Figure 8:**
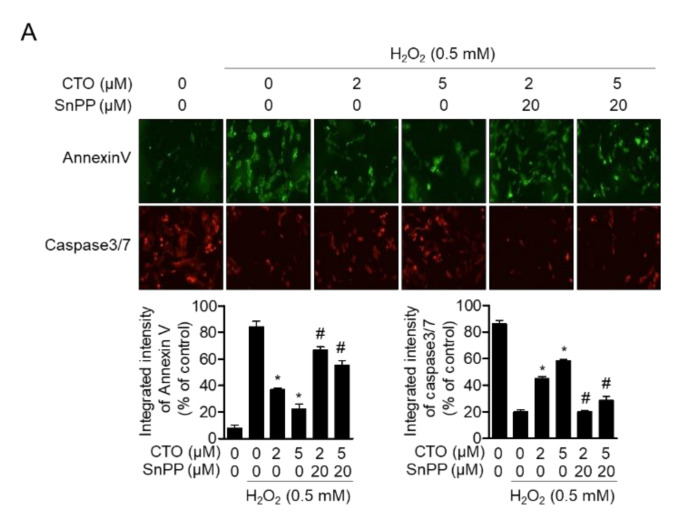
Enhanced HO-1 by CTO shows a protective effect from apoptosis induced by H_2_O_2_ in SW1353 cells. (**A**) SW1353 cells were pre-treated with or without 20 μM SnPP for 1h. Then cells were treated with the indicated concentration of CTO (2 or 5 μM) for 6 h, and incubated with 0.5 mM H_2_O_2_ for 2 h. Apoptotic cells were evaluated by staining with caspase 3/7. Reagents using IncuCyte imaging system. ^*^ *p* < 0.05 vs. only H_2_O_2_ treat group; ^#^ *p* < 0.05 vs. only CTO treated group.

In Section 3.7, we confirmed that CTO protected SW1353 cells H_2_O_2_-induced apoptosis. To investigate whether induced HO-1 expression by CTO pretreatment plays a protective role in apoptosis induced by H_2_O_2_ treatment, the expression of annexin V and caspase 3/7 were assessed in cells treated with SnPP. Suppression of Annexin V by CTO pretreatment was significantly increased by SnPP treatment. The level of caspase-3/7 upregulated by CTO treatment was re-inhibited by SnPP treatment (Figure 8). These results suggest that the induction of HO-1 by CTO pretreatment prevents H_2_O_2_-induced apoptosis in SW1353 cells_._

## 4. Discussion

Promoting the expression of antioxidant enzymes in articular chondrocytes and inhibiting oxidative stress has been considered as a potential therapeutic approach in OA [25,26]. Hydrogen peroxide (H_2_O_2_) is a general type of ROS, and enzymes such as SOD, CAT, GPX, glutathione reductase (GR), and HO-1 protect the oxides caused by oxidative stress [27]. Nrf2 is a nuclear transcription factor that promotes the expression of antioxidant-related enzymes, such as HO-1, through binding to antioxidant response elements (AREs) and shows a protective role against cellular damages [28]. The MAPK pathway is involved in the translocation of Nrf2 to regulate oxidative stress [29]. Western blot analysis showed that CTO increased HO-1 expression depending on the concentration and time of translocation of Nrf2 to the nucleus. Moreover, CTO enhanced the phosphorylation of MAPKs in a time-dependent manner. These results suggest that CTO defends the cells against oxidative damage by up-regulating HO-1 expression via the MAPK pathway.

As one of several antioxidant mechanisms to prevent ROS-induced damage, SOD and CAT play vital roles in reducing oxidative stress by removing H_2_O_2_ [30]. In these mechanisms, CTO down-regulated the level of ROS stimulated by H_2_O_2_, and Western blot analysis showed the recovery of expression levels of antioxidant enzymes previously inhibited by H_2_O_2_. Reduced expression of Col2A1 and Aggrecan in chondrocytes is an important feature of cartilage degeneration; MMP-3 and MMP-13 are responsible for the degradation of the extracellular matrix that damages cartilage structures and properties, and TIMPs inhibit activities of these MMPs [31,32]. Inhibition of MMPs is recognized as a major treatment strategy to block joint cartilage loss in OA, and H_2_O_2_ promotes the expression and secretion of enzymes such as MMP-3, MMP-13, and ADAMTS [33]. CTO effectively inhibited the MMP-3, MMP-13, and ADAMTS enzymes secreted from H_2_O_2_ stimulated chondrocytes, and recovered Col2A1, Aggrecan, and TIMP mRNA, which play an essential role in cartilage degeneration caused by H_2_O_2_. From the previous results, CTO is an HO-1 inducer. Therefore, to investigate the protective effect of CTO-induced HO-1 expression on the H_2_O_2_-stimulated chondrocytes, we reversed the protective effect of chondrocytes by treatment with SnPP, an HO-1 inhibitor.

H_2_O_2_ causes apoptosis by increasing the production of ROS and destroying the mitochondrial membrane potential, which activates Bax of apoptosis and inactivates antiapoptosis Bcl-2 [34,35]. It also sequentially activates stimulators of apoptosis, such as caspase-3 and 7 [36]. In this study, CTO recovered the mRNA and protein expression levels of the anti-apoptotic protein BCL-2 in H_2_O-treated conditions and further inhibited those of apoptotic stimulants, such as BAX, induced by H_2_O_2_. The protein and mRNA levels of caspase-3 were also reversed. Previous studies showed that hypoxia leads to the formation of ROS, which induces oxidative stress and partially activates the transcription factor Nrf2, resulting in HO-1 expression and hypoxia-inducible factors through the phosphoinositide 3-kinase (PI3K)/Akt signaling pathway [37]. In this study, H_2_O_2_-stimulated SW1353 chondrocytes were treated with SnPP, an HO-1 inhibitor, to suppress HO-1 expression induced by CTO. Therefore, the ROS inhibitory and anti-apoptotic effects of CTO as shown above, and the expression of antioxidant enzymes, were reversed. These results suggest that the protective effect on chondrocytes appears through HO-1 expression by the activity of Nrf2 by CTO rather than by Nrf2 activity by H_2_O_2_. These results suggest that, through activation of Nrf2 and HO-1, CTO could suppress ROS production and apoptosis as well as regulate antioxidant enzyme expression stimulated by H_2_O_2_ in SW1353 cells.

## 5. Conclusions

In this study, we investigated the effects of CTO on apoptotic, antioxidant enzymes and cartilage-specific proteins caused by ROS, a major cause of osteoarthritis in H_2_O_2_ stimulated SW1353 cells. We found that CTO effectively regulated ROS generation by H_2_O_2_ and apoptosis of SW1353 through Nrf2/HO-1 activity, and recovered lost antioxidant enzymes. This study revealed a new pharmacological effect of prenylated xanthones CTO, isolated from *M. tricuspidata*, and suggests its potential as a novel natural treatment for osteoarthritis.

## Figures and Tables

**Table 1:** Primer sequences.

Target Gene	Sequence	
*col2a1*	Forward (5′-3′)	TGGACGCCATGAAGGTTTTCT
	Reverse (3′-5′)	TGGGAGCCAGATTGTCATCTC
*aggrecan*	Forward (5′-3′)	GAAGTGGCGTCCAAACCAA
	Reverse (3′-5′)	CGTTCCATTCACCCCTCTCA
*timp1*	Forward (5′-3′)	AATTCCGACCTCGTCATCAG
	Reverse (3′-5′)	GTTGTGGGACCTGTGGAAGT
*mmp3*	Forward (5′-3′)	GGT GTG GAG TTC CTG ATG TTG
	Reverse (3′-5′)	AGC CTG GAG AAT GTG AGT GG
*mmp13*	Forward (5′-3′)	TCA GGA AAC CAG GTC TGG AG
	Reverse (3′-5′)	TGA CGC GAA CAA TAC GGT TA
*adamts*	Forward (5′-3′)	TTCCACGGCAGTGGTCTAAAG
	Reverse (3′-5′)	CCACCAGGCTAACTGAATTACG
*sod*	Forward (5′-3′)	GGGAGATGGCCCAACTACTG
	Reverse (3′-5′)	CCAGTTGACATCGAACCGTT
*cat*	Forward (5′-3′)	ATGGTCCATGCTCTCAAACC
	Reverse (3′-5′)	CAGGTCATCCAATAGGAAGG
*bcl2*	Forward (5′-3′)	AGG CTG GGA TGC CTT TGT GG
	Reverse (3′-5′)	GGG CAG GCA TGT TGA CTT CAC
*bax*	Forward (5′-3′)	TTCTGACGGCAACTTCAACTGG
	Reverse (3′-5′)	AGGAAGTCCAATGTCCAGCC
*caspase3*	Forward (5′-3′)	GGAATTGATGCGTGATGTT
	Reverse (3′-5′)	TGGCTCAGAAGCACACAAAC
*gapdh*	Forward (5′-3′)	ACCCAGAAGACTGTGGATGG
	Reverse (3′-5′)	CACATTGGGGGTAGGAACAC
